# Exploring motivation to notify and barriers to partner notification of sexually transmitted infections in South Africa: a qualitative study

**DOI:** 10.1186/s12889-018-5909-4

**Published:** 2018-08-06

**Authors:** Julia M. Wood, Jane Harries, Moira Kalichman, Seth Kalichman, Koena Nkoko, Catherine Mathews

**Affiliations:** 1 School of Public Health and Family Medicine, University of Cape Town, Observatory, Cape Town, Western Cape 7925 South Africa; 2 Women’s Health Research Unit, School of Public Health and Family Medicine, University of Cape Town, Observatory, Cape Town, Western Cape 7925 South Africa; 3 Department of Psychological Sciences, University of Connecticut, 2006 Hillside Road, Storrs, CT 06269 USA; 4 City of Cape Town, City Health Department Cnr NY 1 Lansdowne Road Fezeka Administration Complex Guguletu, Cape Town, Western Cape South Africa; 5 South African Medical Research Council, Tygerberg, P.O Box 19070, Cape Town, Western Cape 7505 South Africa

**Keywords:** Partner notification, Sexually transmitted infections, South Africa

## Abstract

**Background:**

This article will review qualitative data from intervention-based counselling sessions to explore barriers to partner notification (PN) for South African men and women who have contracted sexually transmitted infections (STIs). This qualitative study took place in a township where there is high STI and HIV prevalence. In addition to reviewing barriers to PN, the study will also identify participants’ perceptions about effective PN strategies that are presented during the intervention. Ultimately, the study will assess the intervention’s impact on participants’ motivation and skills to notify their partners about their STI status.

**Methods:**

Relying on recorded counselling sessions from an intervention run by a parent study, this sub- study reviewed 30 transcripts from counselling sessions with 15 men and 15 women. The intervention was a 60 min interactive session where STI and HIV education, risk mitigation, and effective PN strategies were discussed. Participants were between 19 and 41 years old (mean age = 28.4) and lived within the catchment area of a South African township. Recordings were chosen based on verbal responsiveness of the participant and were manually coded for analysis. In addition, two programme counsellors were interviewed about their perceptions of the intervention and their experiences with participants to enhance rigour and reduce potential bias.

**Results:**

By the conclusion of the intervention session, both male and female participants were motivated to notify their partners face-to-face about their positive STI status. Despite this, misperceptions about the etiology and transmission of STIs, as well as inadequate support from the clinical level and power imbalances amongst men and women emerged as major barriers for the prevention of future STIs.

**Conclusions:**

While the intervention appears to be successful in facilitating partners’ intentions to notify, the data shows significant social and structural barriers that will create difficulties for the prevention of future STIs. Participants’ persistent concerns about acquiring HIV or their current positive status affect decision-making and therefore, could be a window of opportunity for health-care providers or lay counsellors to discuss STIs in high prevalence areas.

## Background

South Africa has one of the world’s largest burdens of HIV infection, which is accompanied by high rates of other sexually transmitted infections (STIs) [1, 2]. Patients with STIs face an increased biological risk of HIV acquisition because of the virus’ invasion of the immune system through genital lesions and/or inflammation caused by STIs [3]. Furthermore, STIs are known to cause a range of complications for patients in their own right including infertility, neonatal conditions for the newborns of STI-infected women, ectopic pregnancies, and certain cancers [4]. There are also social consequences to these infections, particularly for women who are not only more likely to be asymptomatic, but are also more likely to struggle with poor access to health-care, stigmatising and judgmental attitudes from society and health care providers, and intimate partner violence [5].

One of the most effective mitigators of on-going STI transmission is partner notification. Partner notification (PN) constitutes a range of methods for informing index patients’ sexual partner(s) about their STI exposure, followed by encouraging those partners to accept treatment; these methods can include notification directly from the index patient or via the clinician who diagnoses the infection [6].

Like many middle and low income countries, index patients in South Africa often bear the sole responsibility of notifying their partners about their STI; significant barriers to successful notification often complicate this responsibility. For example, studies from South Africa have shown that significant structural barriers exist, which include poor or even absent counselling about infections, minimal training in disease management and counseling, and providers who are unaware about the standards of care that positively diagnosed patients require [7, 8]. In addition, studies examining the cultural context of STI diagnosis in marginalized communities (including townships in South Africa) have identified personal barriers to PN including stigma from communities and fear of blame or violence amongst females who notify their partners [9–12].

Research from various settings has suggested that PN can be best facilitated through single session interventions in which a nurse or lay counsellor explains the importance of PN as an effective strategy for interrupting forward transmission of pathogens and protecting one’s sexual network [13, 14]. Despite this finding, there are few studies that examine HIV prevention via PN counseling in South Africa and in other high prevalence/resource poor settings. This qualitative study will therefore address a current gap in the literature about this area by reviewing transcripts from enhanced counselling intervention sessions that were conducted during a parent study.

The parent study sought to analyse PN outcomes between three groups receiving different counselling packages in a South African township. The objective of the parent study was to measure the outcomes of PN practice after participants received enhanced counselling sessions emphasizing its importance; the parent study compared these participants’ PN outcomes with two groups that did not receive the same intervention. The purpose of this qualitative sub-study was to explore how participants perceived PN, particularly regarding barriers to effective notification. In doing so, this study assessed the impact of enhanced counselling sessions on participants’ motivation and perceived skills to notify their partners about their STI.

## Methods

### Parent study

The parent study of this sub-study was a three arm randomized trial where participants were allocated to three different counselling interventions of varying intensity. These arms were offered one-on-one sessions including an enhanced standard of care group that received a 20 min STI and HIV education session, a group that received STI and HIV education as well as information regarding risk reduction, and an intervention group that received a 60 min educational and motivational enhanced session regarding STI and HIV education, risk mitigation, and effective PN strategies. The intervention arm utilised flip charts, trained counsellors, and interactive activities to underscore its lessons and to build participants’ PN and communication skills. The theoretical framework for the intervention was the Information Motivation Behavioural Skills model of health behaviour change, which emphasizes HIV prevention and motivates participants by presenting risk reduction strategies.

### Setting and inclusion criteria

In order to participate, trained recruitment staff from the parent study would verify that the participant was above the age of 18 and living within the study’s catchment area. The study’s catchment area was an impoverished township in an urban setting in South Africa. Individuals were excluded from participating in the study if they were the known partner of an index patient or if they had tested positive for HIV during their current visit; these provisions were meant to preserve data integrity and to fulfil the ethical obligation of causing no harm to participants.

The South African Department of Health’s guidelines regarding STI management are based on syndromic management in which individuals are treated for most STIs according to their symptoms rather than laboratory confirmation of specific pathogens [15]. Thus, participants were referred to participate in the parent study based on the nurse’s diagnosis of an STI and recommendation for STI treatment at the clinic where the parent study was taking place. From there, participants were recruited and upon providing their consent to participate, they were screened to meet the parent study’s inclusion criteria.

For quality assurance purposes, the parent study’s enhanced counselling sessions were recorded. These intervention sessions included an interactive discussion about the participant’s knowledge of STIs, image guided discussions about particular STIs and their routes of transmission, and finally, an exploration of the participant’s personal sexual network. At this point, the participant was invited to consider communication strategies for notifying his/her sexual partner(s) about receiving treatment for an STI. Participants were offered choices of several notification methods including face-to-face notification, notification via a letter or text message, or notification directly from the clinic. The intervention sessions were conducted by female lay counsellors who were trained by supervisors from the parent study. Topics discussed in the 60 min sessions included addressing misconceptions about STIs, discussing participants’ personal history with STI testing, diagnosis, and treatment, reviewing how to avoid STIs in the future (including discussions about condom usage and examining one’s sexual network), and exploring participants’ realistic notification options and next steps. Participants’ responses for each topic (including their questions to the counsellors) were coded for analysis.

### Substudy: Review of intervention sessions

Initially, this sub-study randomly selected 30 recordings from the 60 min enhanced counselling sessions which were undertaken by trained lay counsellors during the parent study between 2014 and 2016; this selection process rendered an inadequate sample because they included sessions that were cut short (denoted by recordings that lasted 30 min or less) or more frequently, they included sessions where the participant was verbally unresponsive (denoted by the counsellor speaking for the majority of the session). At this point, the primary author individually reviewed 230 enhanced session recordings and purposively selected 30 of those sessions based on unreserved verbal interaction between the interviewer and the participant. Because of expected homogeneity in the socioeconomic conditions of the participants as well as in the number of partners each participant would have (according to his/her gender), balance in the ratio of male to female participants was the only other consideration for purposive selection. The counselling sessions were all conducted in a local language.

Transcripts from 15 men and 15 women’s counselling sessions were translated by an external, accredited translator from isiXhosa to English, transcribed, and analysed. In addition, the study’s two counsellors were included via one-on-one interviews with the primary author; the purpose of these interviews was to triangulate participants’ responses and to enhance analysis. The transcripts were manually coded; the primary author reviewed transcripts in their entirety, identified prominent themes, and coded and categorised the responses within those themes. Codes were derived from the data based on emergent themes and were eventually grouped under three major themes followed by sub-themes. In order to enhance rigour in analysis, the secondary authors reviewed and commented on the codes and theme categorisation. All personal identifiers were changed in order to enhance confidentiality and anonymity. Informed consent for the sessions to be conducted and recorded was obtained through the parent study; interviews with counsellors were preceded by signed informed consent. Ethics approval was obtained from the University of Cape Town’s Human Research Ethics Committee.

## Results

The study found that most participants were motivated to notify their main partners about their STI and believed that their partners would attend a clinic visit. In addition, the study found that HIV status/concerns about infection were critical facilitators of PN intentions, while significant barriers to PN included health education, health system, and interpersonal barriers.

Males were more likely to have concurrent partnerships with more than one partner; this was an inhibiting factor for notification because male participants intended to notify their casual and/or anonymous partners less frequently. Women were also motivated about notifying their partners, however their reasoning for doing so was often explained as a matter of practicality: they had previously notified their partners about an STI, they were concerned about their health generally, or most frequently, they knew that their partners had other partners and understood how notification could prevent re-infection. The ages of the participants ranged between 19 and 41 (mean = 28.4).

Both male and female participants were most likely to select a face-to-face method of notification. Few men opted for other notification methods, however some male participants requested phone calls or clinic intervention in order to reinforce their face-to-face notification method or because their partner was in a different province. Almost every woman in this study intended to notify her main partner face-to-face. Few exceptions to this included women who sought additional support from the clinic in order for their partner to take face-to-face notification more seriously. Several women opted for a phone call or text message as their method of notification for casual partners. Generally, the professed preference for face-to-face notification correlated with participants’ motivation to notify.

In some sessions, participants were either unclear about their notification intentions or did not follow the session’s activities due to interruptions raised by the participant. A total of 5 participants (2 women and 3 men) did not need to notify their main partners because the partners had already attended a clinic visit.

### Effect of HIV status on PN intentions

Concerns about HIV acted as an important motivation for notification. A frequently cited belief was that untreated STIs would become HIV. As a 30-year-old female participant explained:

*If you have an STI in your body, like if you don’t treat it, it causes you to be infected with HIV- if it has been there for a long time without being treated, if you just left it like that.*

While participants’ HIV status was not explicitly solicited, participants often revealed their positive HIV status with counsellors during their intervention session; participants’ HIV status was reflective of high incidence areas in South Africa. For participants who professed a positive HIV status, there was frequent concern about staying healthy and protecting themselves. As one 31- year-old woman expressed:

*If he keeps on giving me these things, I will end up sick or the antiretrovirals are not going to work. Tomorrow STI. Tomorrow STI…. I warned him that… I will go to the police, because I don’t want it with my health... because I know I am [HIV] positive and I want to keep myself safe from all these things.*

Alternatively, for those who were HIV negative, there was a commonly discussed fear of contracting the virus that acted as a motivation for both taking STI treatment and for notifying one’s partner about the STI. Thus, concerns about one’s positive HIV status or fears of acquiring the virus through risky sexual behaviour including untreated STIs facilitated PN amongst participants.

While data from the intervention highlighted participants’ notification intentions, they also showed significant social and structural barriers that will create difficulties for the prevention of future STIs. Three categories were identified as potential barriers to notification: health education, health system, and interpersonal barriers.

### Health education barriers

Participants rarely identified how an individual can contract an STI, but rather, relied on gendered beliefs to explain transmission. For example, STIs were frequently cited as being the woman’s fault, either because of her behaviour during intercourse or because of menstruation.

As a 26-year-old male participant explained:

*She was just finishing her period; after having sex with her, I saw blood. So I just added those things together. She was on her period and there is this dirty thing [the STI]. I looked at that information and thought: she gave this to me intentionally.*

Beliefs about women as STI carriers also stood out to the counsellors who found debunking these beliefs for men to be particularly challenging. In order to do so, counsellors often tried to shift participants’ attention from who was responsible for transmission to how the participant can interrupt forward STI transmission.

Additional beliefs about STIs were that they were either self-generating or the result of poor hygiene. In addition, misperceptions about STI etiology were sometimes based on various disparate, but localised concerns such as witchcraft, tuberculosis diagnosis or medication, or shared public toilets in or around the township community.

### Health system barriers

During their counselling session, many participants described experiences of inadequate or minimal support from the clinic during their most recent visit. This included receiving incorrect information, not receiving any instruction about condom usage or PN after a positive STI diagnosis, and/or receiving medication or tests for which participants were unaware of their purpose. These experiences of inadequate support exclude individuals who- even after having met with the nurse to get treatment- still had limited understanding about STIs. Experiences ranged from moderate examples where participants felt inconvenienced or stigmatized to extreme examples where participants were given incorrect information.

One 30-year-old female participant’s summary of her experience with a clinician showcases various challenges including a negative attitude from the nurse, poor counselling, and insufficient communication about how to promote positive health seeking behaviours in the future:

*Participant: The Sister didn’t say much. She was not in a right mood. She gave me an injection and gave me pills. I didn’t feel right because if you are a person who talks to people, you must be in a right mood, but she...*

*Counsellor: Didn’t she say anything about using a condom?*

*Participant: No, she didn’t say anything. She just gave me pills. She asked what I came for. I said I came for a pap smear. She said I cannot do a pap smear because I did it last year. I kept quiet. I said okay. She said [again] why did you come now? I said, I can feel it underneath that I have an itch... She said you must go and come with your partner tomorrow. I left.*

More extreme examples of inadequate support included judgmental attitudes and/or misinformation from the clinical staff. As a 19-year-old male participant highlighted:

*What I was told about having an STI is that it is wrong. If you leave it inside you for a long time, it will damage you. What will happen is that you will have your private part removed. That’s what I was told by the nurse there.*

Despite this range of experiences, many participants found clinic visits to be inevitable and necessary, particularly for receiving treatment. Female participants expressed limited resistance about going to the clinic in order to address their symptoms quickly, while male participants more frequently described delaying their visit to the clinic and instead, opting for more informal treatments provided at pharmacies or by their friends or partners.

### Interpersonal barriers

Specific interpersonal barriers to notification were most commonly fear of stigma from partners or the surrounding community, concerns about being accused of infidelity, and/or concern about violent reactions from partners. One-on-one interviews with the counsellors revealed similar concerns. As one counsellor stated:

*The main thing that I’ve noticed … when it comes to informing the partners: it’s stigma. ‘People are going to think that I’m cheating. People are going to think that I have multiple partners.’*

Stigma was perceived as a barrier because of generally stigmatised attitudes about sex, the concern about people finding out about the infection in the enclosed township community, and the aforementioned concern that STIs become HIV.

Accusations about infidelity and concerns about violent reactions from partners were cited by men and women, although women were more likely to anticipate the accusations or threats. Some men were unabashed about blaming their female partners for the infection, even after acknowledging their personal sexual risk behaviours. As one 32-year-old man explained:

*She will not be afraid when I say, ‘you must go [to the clinic].’ She knows I can beat her. I will tell her that I got [this infection] from her. ‘That means you go with some dirtiness. Let‘s go to the clinic whilst it is still early.’*

Men were frequently described by female participants as being difficult or stubborn, which complicates women’s ability to alter risk behaviours and/or effectively discuss PN. These circumstances highlight an interpersonal power dynamic that men appear to hold over their female partners. Despite this, many participants understood the importance of their partner receiving treatment and would not be dissuaded from PN. As a 24-year-old female participant explained:

*He can insult me and what-not, but even if that happens, I don’t have a problem with that. As long as I have told him ‘Okay listen brother, you must go to the clinic. You will tell them that you have an STI. So please go.’ And I think he will go.*

Overarching trends are important for contextual insight. The majority of both female and male participants discussed concurrency as being a consideration for PN. For women, awareness of their male partners’ concurrent relationship(s) was often accepted as inevitable and negotiating condom use could be perceived as a challenge. Men were often confident that notification would either be received as symbolic of their care for their partners or that their partners would attend a clinic visit without protest; for this reason, they were often open to notifying most of their reoccurring sex partners about the STI. Both men and women frequently cited drug and alcohol use as contributing to sexual risk behaviours.

### Impact of the intervention

Overall, this intervention was helpful in informing participants about STIs and the importance of PN. Evidence for this was found in several participants’ specific comments about how the intervention changed their way of thinking and/or how they felt encouraged to use barrier methods of protection with fewer partners. As a 24-year-old male participant explained:

*I learned a lot; I learned some things I didn’t know. And other diseases we discussed here have never occurred to me before. I am going to try to avoid them totally. I wouldn’t have known those things and I would neglect them. But now I know what caused this in me and that if this happens, I must go to the clinic.*

Women were generally more expressive about how the intervention affected their way of thinking. Often aware of their partners’ concurrent relationships, numerous female participants were interested in the session’s individualized exploration of their sexual network. This often concluded with a commitment to PN and barrier methods for protection. As a 32-year-old female participant concluded:

*It depends on him if he tells his other partners [about the STI], but I will not have sex with him without a condom.*

While the intervention was often perceived to be informative, sessions frequently lasted less than 60 min, were interrupted by participants, or were dominated by the counsellors; this warrants an adaptation to the sessions’ curriculum.

## Discussion

The findings from this study highlight that health education, health system, and interpersonal barriers can mitigate effective PN. Commonly cited barriers to PN across the literature such as perceived stigma from society, providers, or STI patients themselves [16–18], inability or unwillingness to contact casual or anonymous partners [19], and anticipated violent reactions from male partners [12] were also found amongst participants in this sample. The findings from this research highlight that while interpersonal concerns can be anticipated by patients, misconceptions about STIs and inadequate support from the clinic are also prevalent in this setting and are indicative of wider health education and health system challenges [8].

In order to facilitate PN, this study recommends additional provider training that promotes STI education and support for infected individuals. In addition, power imbalances between men and women must be addressed in order to reduce stigma, concurrency, and blame on females as being STI reservoirs. In summary, the findings from this study recommend that future interventions address these education and support challenges in a way that accounts for the cross-cutting barriers that were explored in this study.

Single session interventions are commonly cited as effective mitigators of forward STI transmission because of their efforts to include index patients with their health seeking behaviours and to strengthen communication skills for notification [20, 21]. Such interventions have been shown to be successful in resource constrained, high prevalence settings [22]; the findings from this study provide additional support that interventions conducted by trained lay counsellors can encourage STI-infected individuals’ intentions to notify their partners about their STI because of their increased understanding of the infection and strategies for notification. According to this study’s data (which were triangulated between existing literature, recordings from counseling sessions, and interviews with counselors), enhanced counselling sessions were often perceived positively. The parent study’s findings are necessary for reviewing the feasibility of the detail and length of enhanced sessions compared to other methods of delivery. Nevertheless, the findings from this sub-study add to the literature which suggests that single session interventions should be prioritised as an enabling pathway for effective PN practices to take place [23].

There are several limitations to this study design. Selection bias may have impacted the results of this study because participants were purposively rather than randomly selected in order to maximise the study’s resources; the absence of data from less responsive participants does not allow for insight on less engaged participants’ intentions and/or barriers regarding PN. While having more verbally responsive participants could have enabled reporting bias, this was a study of intentions; the results of the parent study will be critical for evaluating the success of PN in practice. In addition, there were some inconsistencies in the data that would have benefitted from follow-up questions and additional counsellor training, however as secondary data collection, this study was unable to facilitate those benefits. Finally, the findings from this study may only be a reflection of an urban South African setting; still, the findings could impact similar settings because of their emphasis on structural and interpersonal barriers to notification.

## Conclusions

This study found that South Africa’s generalised HIV burden can be a facilitator to PN of STIs. HIV positive participants were concerned about their well-being and the intervention was a useful learning opportunity for health promotion. HIV negative participants were concerned about maintaining their status, which was influenced by their new understanding of STIs. Overall, widespread preliminary understanding and framing of HIV and how it is transmitted within this high incidence community is a window of opportunity for health workers to discuss other STIs.

This study contributed to a gap in local literature by highlighting how challenges in health education, the local health system, and interpersonal relationships remain for PN of STIs. The information delivered during this intervention challenges interpersonal power balances between partners and encourages participants to be more included in their health seeking and in the health system; this is evidenced by the finding that most participants intend to notify their partners about their STI using a face-to-face notification method. Future efforts to mitigate high STI rates should be cognizant of these considerations. Single session interventions can be effective in facilitating PN, particularly when they use familiar concepts and terminology that patients already understand because of their awareness about HIV in their homes and/or communities.

## Abbreviations

PN: Partner Notification
STI(s): Sexually transmitted infection(s)
